# Preparation and Bolometric Responses of MoS_2_ Nanoflowers and Multi-Walled Carbon Nanotube Composite Network

**DOI:** 10.3390/nano12030495

**Published:** 2022-01-31

**Authors:** Qin Wang, Yu Wu, Xin Deng, Liping Xiang, Ke Xu, Yongliang Li, Yangsu Xie

**Affiliations:** College of Chemistry and Environmental Engineering, Shenzhen University, Shenzhen 518055, China; WQ1910223067@163.com (Q.W.); 2017145023@email.szu.edu.cn (Y.W.); dengxin2017@email.szu.edu.cn (X.D.); xlp727104513@163.com (L.X.); xkenergy@hotmail.com (K.X.); liyli@szu.edu.cn (Y.L.)

**Keywords:** CNT network, MoS_2_ nanoflowers, bolometer, uncooled, photothermal performance

## Abstract

Due to their broadband optical absorption ability and fast response times, carbon nanotube (CNT)-based materials are considered promising alternatives to the toxic compounds used in commercial infrared sensors. However, the direct use of pure CNT networks as infrared sensors for simple resistance read-outs results in low sensitivity values. In this work, MoS_2_ nanoflowers are composited with CNT networks via a facile hydrothermal process to increase the bolometric performance. The thermal diffusivity (*α*) against temperature (*T*) is measured using the transient electro-thermal (TET) technique in the range of 320 K to 296 K. The *α*-*T* curve demonstrates that the composite containing MoS_2_ nanoflowers provides significant phonon scattering and affects the intertube interfaces, decreasing the *α* value by 51%. As the temperature increases from 296 K to 320 K, the relative temperature coefficient of resistance (TCR) increases from 0.04%/K to 0.25%/K. Combined with the enhanced light absorption and strong anisotropic structure, this CNT–MoS_2_ composite network exhibits a more than 5-fold greater surface temperature increase under the same laser irradiation. It shows up to 18-fold enhancements in resistive responsivity ((*R*_on_ − *R*_off_)/*R*_off_) compared with the pure CNT network for a 1550 nm laser at room temperature (RT).

## 1. Introduction

Fast and sensitive infrared (IR) detectors operating at room temperature are of tremendous interest for industrial, scientific, and military applications, including in security, environmental monitoring, remote controls, optical communication, thermography, and astronomy, as well as for the latest technologies, such as in self-driving cars and for obstacle avoidance in robots [1,2,3,4,5]. Traditional bolometers consist of an absorber and a sensor. During detection, thermal radiation is absorbed by the absorber, leading to a temperature increase, subsequently resulting in a change in electrical resistance in the sensor, which can be measured using electrical circuits. Then, through electrical signal processing, the temperature of the target object is obtained. Nowadays, the main commercial uncooled thermistor materials are amorphous silicon (a-Si), vanadium oxide (VO_2_), and germanium–silicon–oxide [6,7,8]. However, a-Si shows long response times of tens to hundreds of ms [9,10]. The production of VO_2_ causes great environmental pollution [11,12]. Furthermore, the commercialized uncooled bolometers require sophisticated designs such as micro-bridges or thermal insulation layers to obtain good thermal insulation [13,14], as well as an extra IR absorption layer [15] to ensure good photon absorbance. Thus, although high performance can be achieved with the above complex designs, more accessible uncooled bolometric materials with self-absorbing, self-thermal-insulating, and self-sensing properties are in great demand to increase the application of bolometers in real life.

Due to their broadband IR absorption and fast responses of up to picoseconds resulting from the ultrahigh carrier mobility and weak electron–phonon scattering, carbon nanotubes (CNTs) and their composites have attracted wide attention as some of the most promising candidates for flexible IR detectors [16,17,18,19,20]. However, the TCR (temperature coefficient of resistance) of CNTs is low, which makes simple resistive read-outs difficult. Itkis et al. reported a large bolometric photoresponse of suspended single-walled CNT (SWCNT) films with TCR values of 1% at 330 K and 2.5% at 100 K [21], which were close to those of a VO_2_ bolometer [22,23]. However, the large-scale production of single-walled carbon nanotubes (SWNTs) of high quality and purity is expensive and challenging, which limits their application [24,25]. The relatively cheaper price of MWCNTs and compromised but still excellent optical, electrical, and mechanical strength makes them a good candidate for bolometer applications. Randomly assembled MWCNT films synthesized by vacuum filtration is some of the most accessible forms of bulk CNT materials suitable for large-scale production and application. Nevertheless, for MWCNT films, the TCR was reported to be only 0.088%/K [26]. The high *k* and low TCR of pure MWCNT films result in weak temperature sensitivities and lead to poor bolometric performance. It would ideal if CNT bolometers could be used with simple resistive read-outs and could be manufactured without the use of high-quality CNTs or delicate microfabrication processes.

To improve the bolometric performance of CNTs, photothermal materials with high light absorption and TCR can be composited with CNTs such as graphene [17,18] and metal oxides (ZnO, VO_2_) [20,27,28,29]. Lu et al. achieved novel exciton dissociation of a graphene–MWCNT hybrid film through heterojunctions self-assembled at the graphene–MWCNT interfaces. This method significantly improved the responsivity of the CNTs in the near-infrared region [18]. Nandi et al. used a spray coating method to prepare a suspended bolometer based on an MWCNT coated with vanadium oxide. The suspended bolometer showed a high TCR of ~−0.41%/K, which was ~4.86 times higher than that of the previously reported suspended MWCNT film [22]. Recently, it was reported that MoS_2_ with a flower-like or spiral-like shape showed excellent light absorption performance [30,31,32]. Tahersima et al. reported on the rolling of Van der Waal heterostructures of molybdenum disulfide (MoS_2_)–graphene (Gr)–hexagonal boron nitride (hBN) into a spiral solar cell, leading to strong light matter interactions and allowing for solar absorption up to 90% [31]. Yang et al. prepared an ultrathin 2D porous film for solar steam generation based on MoS_2_ nanosheets and an SWCNT film. Even at an ultra-thin thickness of about 20 nm, its absorption rate across the entire solar spectrum range exceeded 82% [30]. Thus, it is advantageous for CNTs to be composited with flower-like or spiral-like MoS_2_ to improve the bolometric performance.

In this work, MoS_2_ nanoflowers are composited with a CNT network via a facile self-assembling strategy. The CNTs act as a thermally and electrically conductive network, while the MoS_2_ nanoflowers not only enhance the broadband absorbance, but also influence the intertube coupling in the CNT network, resulting in an improved TCR value. The thermal and electrical transport properties over the temperature range of 296 K–320 K are investigated. The figures of merit of the free-standing composite network, including the photothermal performance, resistive responsivity [(*R*_on_ − *R*_off_)/*R*_off_], detection sensitivity to a wide spectrum ranging from ultraviolet to near-infrared, and response times are studied and compared with the pure CNT network in detail.

## 2. Materials and Methods

### 2.1. Preparation of the CNT–MoS_2_ Composite Network

The CNT network was purchased from XFNANO and was prepared from CNT powder by vacuum filtration. A piece of the CNT network with lateral dimensions of about 1 cm × 1 cm was cleaned with N_2_ plasma (200 W, 120 s). Sodium molybdate (Na_2_MoO_4_) and thiourea (CH_4_N_2_S) were dissolved in deionized water with magnetic stirring for 30 min to form precursors with two different suspension concentrations (Table 1). The resulting solutions were denoted solution 1 and solution 2, respectively. Next, the CNT network and the prepared mixture were put into a 100 mL autoclave and reacted at 200 °C for 24 h. The samples were then removed and washed with deionized water and dried in an oven at 60 °C for 12 h. This hydrothermal process can be used to assemble MoS_2_ flakes with flower-like or spiral-like nanostructures in the nm–um size range, which can significantly improve the light absorption performance [30,32,33,34]. Finally, the CNT–MoS_2_ composite network was annealed in a tube furnace at 900 °C under Ar atmosphere for 2 h with a heating rate of 2 °C/min. The reaction routes can be expressed as follows [35]:

Na_2_MoO_4_ 2H_2_O + CH_4_N_2_S + H_2_O → MoS_2_ + NH_3_ + CH_3_COOH + NaOH

### 2.2. Structural Characterization Methods

In order to characterize the micro-structures of this composite network, we used X-ray diffraction (XRD), X-ray photoelectron spectroscopy (XPS), Raman spectroscopy, and scanning electron microscopy (SEM). The SEM images were taken using a JSM-7800F TEAM Octane Plus instrument with a voltage of 10 kV. The XRD spectroscopy was carried out by using an Empyrean diffractometer (PANalytical, the Netherlands) with Cu K*α* radiation (λ = 1.54 Å) at a generator voltage of 45 kV and a generator current of 40 mA. The elemental composition and functional group analysis were tested using a Thermo Scientific K-Alpha XPS instrument. The Raman spectra were obtained using a Horiba LabRAM HR Evolution instrument. The UV–Vis–NIR spectrometer was used to characterize the absorbance of samples in the range of 300–2000 nm. The instrument was equipped with an integrating sphere to measure transmittance (*T*) and total reflectance (*R*), and finally to obtain the absorbance values (*A* = 1 − *T* − *R*).

### 2.3. Characterization of the Thermal Diffusivity and TCR

The transient electro-thermal (TET) technique was used to characterize the thermal diffusivity (*α*) of the samples. The CNT–MoS_2_ composite network was cut into long rectangular strips, then suspended between two aluminium electrodes (the size of the measured samples in this work is presented in Table 2). A small amount of silver paste was used to fix the ends of the strip onto the electrodes and to reduce the contact resistance [1]. Before the measurement, the sample stage was installed on a cold head in a closed-cycle cryostat system (Janis, CCS) where the environmental temperature was controlled from 320 K to 296 K. The environment temperature, provided through the temperature of the cold head of the cryogenic system, was used to for the characterization of the electrical and thermal properties. At the same time a vacuum environment was provided, in which the air pressure was maintained below 10^−2^ Pa. The electrodes were connected in parallel with a current source (Keithley 6221) and an oscilloscope (Tektronix MDO 3054).

During the measurement, a step current was fed to the sample through a current source, causing a small and fast joule heating. Here, a one-dimensional heat transfer model can be assumed reasonably. Within a small temperature range, it can be assumed that the TCR of the sample is constant. Then, the normalized temperature can be obtained from the normalized voltage profile as: T* = V* = (*V*_sample_ − *V*_0_)/(*V*_∞_ − *V*_0_), where *V_0_* and *V_∞_* are the voltage of the sample before the joule heating and when it reaches steady state, respectively. Thus, the averaged normalized temperature T* = [*T*(*t*) − *T*_0_]/[*T*(*t*→∞) − *T*_0_] can be derived as [36,37]:

(1) $$T^{*}=\frac{48}{\pi^{4}}\sum_{m=1}^{\infty }\frac{1-{\left(-1\right)}^{m}}{m^{2}}\frac{1-\exp\left[-m^{2}\pi^{2}\alpha_{measure}t/L^{2}\right]}{m^{2}}$$

where *m* is the normalized parameter, *α_measure_* is the thermal diffusivity of sample, *t* is time, and *L* is the suspended length of the sample. Based on Equation (1), the *α_measure_*can be obtained using MATLAB and via the least squares fitting of the *V*-*t* data. Different trial values of *α* are used for the fitting. The fitting errors were determined to be ±10% or better in our previous work based on the TET technique [36]. During the measurement, *R* is measured using the current source and the oscilloscope in 2-point configurations, with a small bias current (*I*) applied and voltage (*V*) probed. *R* is then calculated by *R* = *V*/*I*. TCR is then obtained by differentiating the *R*-*T* curve.

### 2.4. Test of Bolometric Response

In this process, the composite sample (S2, details shown in Table 2) is suspended between two silicon electrodes using the same method as that described in the TET characterization. Before the photodetection test, the whole sample is installed in a vacuum chamber, whose optical window is made of fused quartz. During the test, the suspended sample is fully covered by the laser spot. The laser power irradiated on the sample is adjusted using the laser output and an optical filter. The laser power is measured using an optical power meter (from Thorlabs company in this study). The power density is calculated by *P*/(πd^2^/4), where *P* is the laser power and *d* is the measured laser beam diameter, as illustrated in Figure S1 in the Supporting Information. The resistance response of the sample is collected using a 7½ digital multimeter (KEITHLEY DMM7510). Upon laser irradiation, the resistances when the laser is turned on and off are denoted as *R*_on_ and *R*_off_, respectively.

### 2.5. Measurement of the Response Time

To measure the transient resistive responses to the lasers, the 405 nm, 860 nm, 1064 nm, and 1550 nm laser outputs are used as the optical sources. The laser outputs are modulated to a 0.2 Hz square wave using a function generator. By applying a small DC current to the sample, with which no appreciable heating occurs, the two-point voltage profiles under the square-wave laser illumination can be recorded using the oscilloscope. In the comparative experiment, to measure the transient resistive response to the joule heating, a square-wave current of 16 mA in amplitude and 0.2 Hz in frequency is applied to the sample using the current source to check the response and to compare it with the response to the modulated laser. The transient resistive response (*V*-*T* profiles) is measured using the oscilloscope. Then, the normalized voltage can be obtained from V* = (*V*_sample_ − *V*_0_)/(*V*_∞_ − *V*_0_), where *V*_0_ and *V_∞_* are the voltages of the sample before the heating or illumination and when it reaches the steady state, respectively. From the V*-*t* curve, the response time is identified when V* is decreased by 0.95.

## 3. Results

### 3.1. Material Synthesis and Structural Characterization

**Figure 1:**
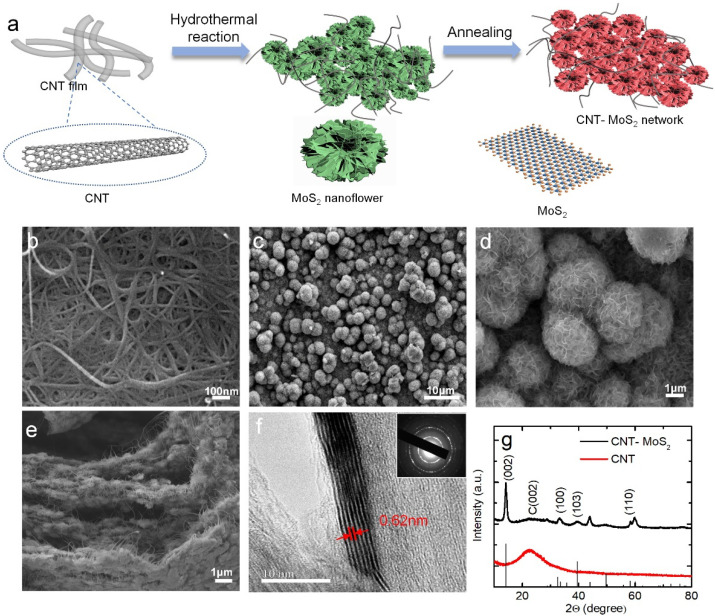
(**a**) Schematic illustration of the synthesis process of the CNT–MoS_2_ composite network. (**b**) The SEM images of the unannealed CNT network. (**c**,**d**) The SEM images of the CNT–MoS_2_ composite network with low to high magnification. (**e**) The SEM of the cross-section of the CNT–MoS_2_ composite network. (**f**) HRTEM images of the CNT–MoS_2_ composite network. The inset shows the SAED pattern. (**g**) XRD spectrum of the unannealed CNT network and CNT–MoS_2_ composite network.

Figure 1a shows the schematic of the synthesis process of the CNT–MoS_2_ composite network. The details can be found in the experimental section. In this process, the N_2_ plasma-cleaned CNT network is placed into the mixture of sodium molybdate and thiourea for hydrothermal treatment at 200 °C for 24 h, then it is thermally annealed at 900 °C in Ar atmosphere for 2 h. The hydrothermal method is chosen to synthesize MoS_2_ because it can assemble the MoS_2_ nanoflakes with different structures in the nm–µm size range. During the process of hydrothermal treatment, the amorphous MoS_2_ nanoflakes grow on the surface and in the interlayer space of the CNT network. As the concentration of the mixture of sodium molybdate and thiourea increases, the shape of the MoS_2_ changes from randomly arranged nanoflakes to a spherical assembly anchored at the surface and in the interlayer space of the CNT network (Figure S2). After the thermal annealing treatment, the amorphous MoS_2_ is transformed into well-crystalized MoS_2_ [30].

The SEM images of the unannealed CNT network and the CNT–MoS_2_ composite network are shown in Figure 1b, c, respectively. From Figure 1b, it can be observed that the diameter of the CNTs ranges from 20 to 30 nm. These tubes are tightly entangled, displaying a randomly packed network [38]. Figure 1c shows the low-magnification SEM images of the CNT–MoS_2_ composite network, where the 3D flower-like MoS_2_ nanoflakes are grown on the surface and in the interlayer space of CNT network [39]. Under SEM at high magnification (Figure 1d), it can be clearly observed that the MoS_2_ flowers with lateral sizes of 500 nm–3 µm assemble with each other [30]. Figure 1e shows a cross-section of the CNT–MoS_2_ composite network, where it can be seen that the carbon nanotubes and MoS_2_ spheres are well combined. The high-resolution transmission electron microscopy (HRTEM) image of the CNT–MoS_2_ composite network is shown in Figure 1f. The low-resolution TEM is shown in Figure S3. The TEM image shows a typical lattice spacing of 0.62 nm, corresponding to the (002) plane of MoS_2_. Four peaks are shown in selected area electron diffraction (SAED) patterns (inset in Figure 1f), which correspond to the (002), (100), (103), and (110) crystal planes of MoS_2_, respectively, indicating the high crystallinity of MoS_2_ [30,40,41].

Figure 1g shows the XRD patterns of the unannealed CNT network and the CNT–MoS_2_ composite network. The pure CNT network only displays a typical diffraction peak at 22.8°, which corresponds to the (002) crystal planes [30,42]. In comparison, the CNT–MoS_2_ composite network shows five peaks at 14.4°, 22.8°, 32.7°, 39.5°, and 58.3°. The diffraction peak at 2θ = 22.8° corresponds to the CNTs [42] and the other peaks can be attributed to the (002), (100), (103), and (110) crystal planes of the hexagonal phase MoS_2_, respectively [30,35,43]. The sharp peaks reveal that MoS_2_ has a well-developed crystalline structure [41]. The (002) d-spacing of MoS_2_ is calculated to be 0.62 nm according to the diffraction peak at 2θ = 14.4° using Bragg*’*s equation, which agrees well with the TEM results [41]. These results demonstrate that the well-crystalized MoS_2_ has been successfully composited within the CNT network.

**Figure 2:**
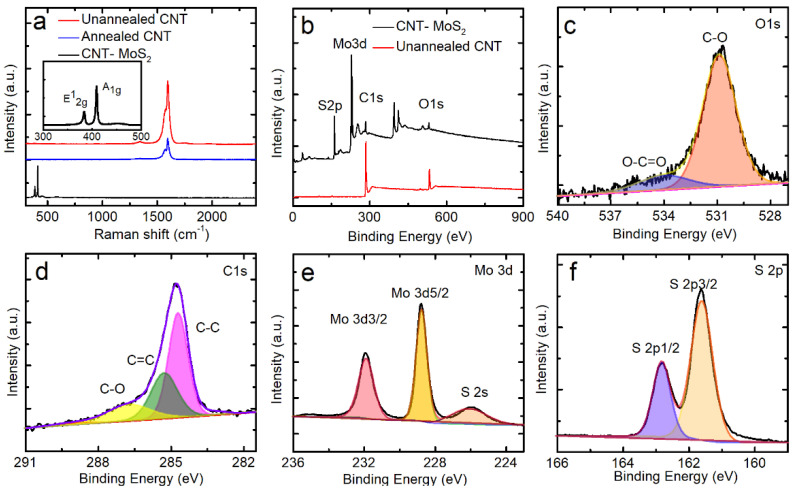
(**a**) Raman patterns of the unannealed and annealed CNT networks and CNT–MoS_2_ composite network. (**b**) XPS survey of the unannealed CNT network and CNT–MoS_2_ composite network, as well as the high-resolution deconvoluted (**c**) O1s, (**d**) C1s, (**e**) Mo3d, and (**f**) S2p spectra of the CNT–MoS_2_ composite network.

The Raman spectra of the CNT network and the CNT–MoS_2_ composite network are shown in Figure 2a, where the pure CNT network shows two pronounced peaks at 1341 cm*^−^*^1^ and 1588 cm*^−^*^1^. The G mode originates from the stretching of the C-C bond, which is usually assigned to zone center phonons of E_2g_ symmetry. The D peak characterizes the disordered degree of the sp^2^ hybrid bond structure in the graphite structure [44,45,46]. The intensity of the D peak to that of the G peak (*I_D_*/I_G_) can be used to estimate the density of disorders of the carbon materials. The *I_D_/I_G_* of the original CNT network is 0.039, while the *I_D_/I_G_* is 0.028 after annealing treatment, which shows a small decrease, indicating that the structure of the CNTs is purified by the thermal annealing process. The full width at half maximum (FWHM) of the original CNT network is 27.2 cm^−1^, which is larger than the CNT network after thermal annealing (22.3 cm^−1^). The low I_D_/I_G_ indicates that the defect density of the original CNT network is low. The thermal annealing treatment further reduces the defects density. After the MoS_2_ deposition, two obvious peaks at 384 cm*^−^*^1^ and 409 cm*^−^*^1^ appear corresponding to the E^1^_2g_ and A_1g_ modes of the hexagonal MoS_2_, respectively. The E^1^_2g_ and A_1g_ modes represent the molybdenum and sulphur atoms displaced in the layer, respectively. The frequency difference between A_1g_ and E^1^_2g_ modes is 25 cm^−1^, indicating that the MoS_2_ in the CNT–MoS_2_ composite network is multi-layered [47,48]. The D peak and G peak of the CNT network cannot be observed using Raman spectroscopy due to the MoS_2_ composite layer covering the surface and the interlayer space of the CNT network.

The elemental composition and functional groups of the CNT–MoS_2_ composite and CNT network are characterized and compared here using XPS. The XPS spectrum of the CNT–MoS_2_ composite network reveals the existence of C, O, Mo, and S (Figure 2b). It can be observed that the intensity levels of O1s and C1s show an obvious weakening trend with the MoS_2_ composite. The main reason for this phenomenon is the large amount of MoS_2_ composite, which is consistent with the Raman spectra results [49]. Figure 2c shows the O1s spectrum, which can be deconvoluted into two peaks at 530.8 eV and 534.0 eV, corresponding to the C-O and O-C=O, respectively. As shown in Figure 2d, the peak of C1s can be deconvoluted into three peaks at 284.8 eV, 285.3 eV, and 286.9 eV, corresponding to C-C, C=C, and C-O, respectively. Figure 2e, f further proves the existence of the MoS_2_. Figure 2e shows a high-resolution Mo3d spectrum with two peaks at 229.1 eV and 232.3 eV. These peaks correspond to the binding energies of Mo3d_5/2_ and Mo3d_3/2_, respectively, and confirm the presence of Mo^4+^ [50]. Furthermore, the weak peak at 226.3 eV is attributed to S2s. The peaks of S2p are located at 161.8 eV and 162.9 eV (Figure 2f), which are related to S2p_3/2_ and S2p_1/2_, respectively. These XPS data further confirm the formation of MoS_2_.

### 3.2. Thermal Properties and Temperature Sensitivity

**Figure 3:**
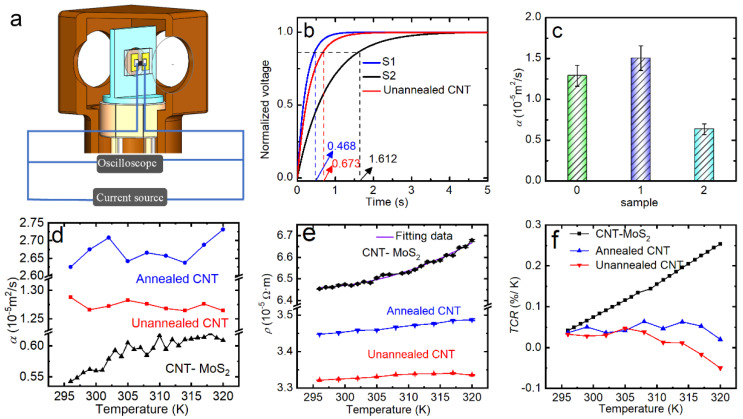
(**a**) Schematic of the experimental setup used for measuring the α and electrical resistance values from 296 K to 320 K. (**b**) The normalized voltage curves of TET signals and the characteristic times of the unannealed CNT network and the CNT–MoS_2_ composite network with low and high MoS_2_ composite density, respectively. (**c**) A comparison of the measured α value at RT against the MoS_2_ composite density. (**d**) The measured α value. The measurement uncertainty of α based on the TET technique is ±10%, which is omitted in the figure for better comparison. (**e**) A comparison of the resistivity and (**f**) TCR values of the samples at different temperatures (296 K–320 K).

To study the effect of the MoS_2_ composite concentration, we prepared samples with different composite concentrations. In this work, the transient electro-thermal (TET) technique was used to measure α values of the CNT–MoS_2_ composite network [36,37,51,52]. For comparison, the pure CNT network after the thermal annealing was also studied. The details for the measured samples are presented in Table 2. Figure 3a shows a schematic of the experimental setup used for measuring the α and electrical resistance (*R*) values of the network using the TET technique. The details can be found in the experimental section.

To study the effect of the MoS_2_ composite concentration, we prepared samples with different composite concentrations, as shown in Table 1, where S1 is the composite network with low MoS_2_ composite density and S2 is the composite network with high MoS_2_ composite density. Figure 3b shows the normalized voltage V* = [V(t*)v− V_0_]/[V_∞_ − V_0_]* versus time for the unannealed CNT network and the CNT–MoS_2_ composite network. According to Equation (1), when the suspended length is constant, the higher α, the shorter the time taken to reach the steady state. It can be seen that the characteristic time (the time when V* reaches 0.8665) [36] of the CNT–MoS_2_ composite network with the high composite density is much longer than that of the unannealed CNT network. The characteristic time of the unannealed CNT network is longer than the CNT–MoS_2_ composite network with the low composite density [36,37,51,52]. As shown in Figure 3c, as the concentration of the MoS_2_ composite increases, the α of the composite network first increases and then decreases. The α increases from 1.29 ± 0.13 × 10*^−^*^5^ m^2^/s for pure CNTs to 1.50 ± 0.15 × 10*^−^*^5^ m^2^/s for S1, which is a 1.2-fold increase. As the MoS_2_ composite concentration further increases, the α decreases to 6.36 ± 0.64 × 10*^−^*^6^ m^2^/s, which is a 51% reduction compared with the unannealed CNT network. Therefore, to ensure a good thermal insulation effect, S2 with the much higher MoS_2_ composite and lower α was chosen for the bolometric performance study, which is denoted as the CNT–MoS_2_ composite network in the following section. The fitting process for these TET signals was conducted using MATLAB. Different trial values of α were used for the fitting. The fitting errors were determined to be ±10% or better, as studied carefully in our previous work based on the TET technique [36].

In order to study the underlying phonon propagation mechanisms, α values of the unannealed CNT network, annealed CNT network, and composite network (S2) were further measured in the temperature range of 320 K to 296 K (the details of the samples in this work are presented in Table 2). As shown in the Figure 3d, for the unannealed CNT network, α decreases from 1.29 × 10*^−^*^5^ m^2^/s to 1.24 × 10*^−^*^5^ m^2^/s when the temperature increases from 296 K to 320 K. This trend is similar to the previous reports for carbon-based materials [37]. However, the α of annealed CNT network increases from 2.62 × 10*^−^*^5^ m^2^/s to 2.73 × 10*^−^*^5^ m^2^/s when the temperature increases from 296 K to 320 K. For the CNT–MoS_2_ composite network, as the environmental temperature increases from 296 K to 320 K, the α value of the CNT–MoS_2_ composite network gradually increases from 5.43 × 10*^−^*^6^ m^2^/s to 6.08 × 10*^−^*^6^ m^2^/s. The unusual α-T behavior of the annealed CNT network and CNT–MoS_2_ composite network indicates that the effect of phonon scattering at intertube interfaces dominates the thermal transport within them [51]. As the temperature goes down, the thermal expansion of the CNTs could deteriorate the contact among CNTs and contributes to the decreasing α. The detailed data for CNT–MoS_2_ composite network can be found in Supporting Information in Figure S4.

Figure 3e and Figure S4 shows the measured *ρ*-*T* curves of the CNT network and the CNT–MoS_2_ composite network. Since the maximum test temperature of the closed-cycle cryostat system (Janis, CCS) can only reach 320 K, we could not obtain data above 320 K. In the future, a new testing chamber will be required to measure the TCR at higher temperatures. As shown in the figures, the resistivity of the unannealed CNT network increases with the rising temperature from 296 K to 315 K, showing a metallic behavior [53]. As the temperature increases from 315 K to 320 K, the resistivity of the unannealed CNT network decreases a little. The resistivity of the annealed CNT network increases monotonously with the increasing temperature. For the CNT–MoS_2_ composite network, the resistivity increases monotonously and nonlinearly with the increasing temperature across the whole temperature range from 295 K to 320 K. The *ρ-T* curve can be fitted well using a quadratic function (Figure 3e). The temperature coefficient of resistance (TCR) is the key characteristic used for evaluating the bolometric performance, which can be calculated using the formula TCR = d*R/*(*dT·R_T_*), where *R_T_* is the resistance at temperature *T*. To reduce the fluctuation of the TCR curve, the Savitzky–Golay function is used to smooth the resistance curves first. As shown in Figure 3f, the TCR of the CNT–MoS_2_ composite network increases with the increasing temperature. At 296 K, the TCR is about 0.03–0.04%*/*K for both the pure CNT network and the composite network, which is consistent with the reported values in the literature [54]. The TCR for the pure CNT network stays around or below 0.05%*/*K in the temperature range of 296 K to 320 K. For the composite network, as the temperature increases from 296 K to 320 K, the TCR increases from 0.04%*/*K to 0.25%*/*K, which is 6 times higher.

Compared with the pure CNT network, the CNT–MoS_2_ composite network shows a metallic electrical resistivity of stronger temperature dependence. As the temperature increases from 296 K to 320 K, the relative TCR of the CNT–MoS_2_ composite network increases from 0.04%*/*K to 0.25%*/*K. From the XPS data, the CNTs are not chemically doped by S. Thus, the existence of the MoS_2_ nanoflowers mainly affects the physical structure of CNT network. The 6-fold higher TCR at 320 K indicates that the thermal strain effect becomes more significant in the electron transport of CNTs due to the MoS_2_ composite, leading to a much higher TCR. For CNT–MoS_2_ composite network, the CNTs play the role of an electrical connecting network. When the temperature changes, the thermal expansion of the MoS_2_ and CNTs is different, which leads to thermal strain on the CNTs. It has been reported that the electrical properties of CNTs are not only affected by intrinsic factors, but also extrinsic factors such as the thermal strain and the significant intertube contact resistance [55,56,57,58,59]. The positive TCR of graphene under strain has been illustrated in the literature [51,60,61]. For CNTs, first-principle calculations have shown that the electronic band structures and the electron–phonon scattering rates are strongly correlated with axial strain [62,63]. However, due to the much larger diameter, the intrinsic conductivity of MWCNTs is expected to be less affected by the strain. The strain can affect not only the intrinsic electrical transport of CNT, but also the intertube interface contact resistance, which could be the main reason for the nonlinear temperature dependence of the network resistance. Liu et al. reported an abnormal temperature coefficient of resistance for PMMA-supported graphene [61]. The combined effects, including the positive thermal expansion of the PMMA, negative thermal expansion coefficient of graphene, and intrinsic resistance change of relaxed graphene against temperature, determined the observed strong nonlinear *R-T* jointly. In our previous work, we found a very strong nonlinear temperature dependence of resistance for ultra-light graphene aerogels, where the interfaces played a dominating role in thermal transport. The strong nonlinear behavior resulted from the temperature-sensitive interconnection among graphene flakes [51]. In the literature, it was found that the temperature coefficient of resistance of graphene nanowall–polymer films changed from around 6%*/*K at 25 °C to 180%*/*K at 40 °C due the thermal strain effect [64]. Thus, the strain effect on CNTs is expected to contribute to the higher TCR of the CNT–MoS_2_ composite network. For the carbon nanotube bolometer, the TCR values at room temperature were found to be about −0.07%*/*K and −0.03%*/*K for 90-nm-thick purified and 100-nm-thick COOH-functionalized SWCNT films, respectively [65]. Lu et al. prepared a SWCNT bolometer with a TCR of 0.17%*/*K and a MWCNT bolometer with a TCR of 0.07%*/*K [19]. Kumar et al. prepared a bolometer based on the MWCNT film with TCR of 0.088%*/*K at RT [26]. Although the TCR of the CNT–MoS_2_ composite network was still lower than that of commercial thermistor materials, the TCR of the CNT–MoS_2_ composite network at 320 K was improved significantly compared to the pure MWCNT films reported in the literature.

### 3.3. Photothermal Performance

**Figure 4:**
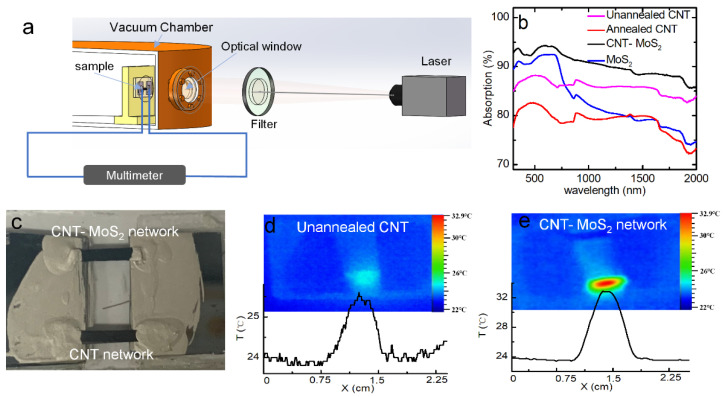
(**a**) Schematic of the experimental setup used for measuring the bolometric response at room temperature. (**b**) Comparison of UV–Vis–NIR absorption spectra for the CNT, MoS_2_, and CNT–MoS_2_ composite networks. (**c**) A photograph of the two suspended samples. Infrared images of the suspended (**d**) CNT network and (**e**) CNT–MoS_2_ composite network under the same uniform laser irradiation. The coordinate axis shows the temperature distribution along the horizontal direction.

The resistive bolometric responses to the laser illumination in ultraviolet to near-infrared wavelength ranges were measured in this work. Figure 4a shows the experimental setup. To compare the light absorbance of the CNT network, for the pure MoS_2_ and the CNT–MoS_2_ composite network, the UV–Vis–NIR spectra characterization was conducted, where the absorption spectra from 300–2000 nm were measured. As shown in Figure 4b, the absorption of the composite network is higher than the CNT network and MoS_2_ powders. The unannealed and annealed CNT networks show absorption in the ranges of 83–87% and 72–82%, respectively, while the MoS_2_ exhibits an absorption range of 74–92%. The MoS_2_ composite increases the absorbance of the CNT–MoS_2_ composite network to 85%-94% over the whole range of 300–2000 nm. Yang et al. [30] also reported that in the wavelength range of 300 nm to 2500 nm, the photon absorption capability of a CNT–MoS_2_ composite network was significantly higher than that of a CNT network, which increased from 40–88% to 90–95%. The main reason for this phenomenon can be attributed to the synergistic photon absorption effect of the MoS_2_ and CNT, as well as the higher thickness of the samples after the MoS_2_ composite.

To investigate the photothermal performance of the composite network, the temperature increases of the CNT network and the CNT–MoS_2_ composite network under the 36.8 mW uniform laser irradiation were measured using an infrared camera (Fotric 227s) in air. The CNT network and CNT–MoS_2_ composite network were cut into rectangular shapes of the same length and width. Then, the two samples were suspended between two silicon electrodes. Silver paste was used to connect the samples with the electrodes. Figure 4c shows a digital photograph of the suspended samples. The infrared images of the samples under 36.8 mW uniform 405 nm laser irradiation as their temperature reached steady state are shown in Figure 4d, e, respectively. The inset figures show the temperature distributions along the horizontal direction for the two samples. It can be seen that at the steady state, the surface temperature of the pure CNT sample increases from 23.8 °C to 25.6 °C, while the surface temperature of the CNT–MoS_2_ composite network increases from 23.8 °C to 32.9 °C. The surface temperature increase for the composite network (9.1 °C) is more than 5 times that of the CNT network (1.8 °C). This can be attributed to the higher photon absorbance and the stronger anisotropic structure of the composite network. According to Figure 4b, it can be seen that MoS_2_ has strong light absorption in the ultraviolet wavelength and gradually weakens in the infrared wavelength, while CNTs can absorb the light in the infrared wavelength. The light is mainly absorbed by the top layer of the network. When the temperature of the top layer is increased as a result of the absorbed light, the thermal energy is then conducted to the electrodes and the bottom layer. In addition, the thermal energy is dissipated through thermal radiation. As can be seen in Figure 1e, the MoS_2_ is composited on the surface and in the interlayer space of the CNT network. As a result, along the thickness direction, the thermal conduction could be greatly impeded by the porous MoS_2_ nanoflowers as well as the resulting interlayer voids. As a result, heat is localized significantly near the top layer of the CNT network. This means the temperature of the top layer is very high, resulting in higher thermal energy loss through thermal radiation. However, the bottom surface shows a smaller temperature rise. Therefore, the amount of thermal radiation from its lower surface is reduced compared to CNT network. It should be noted that since the infrared image was taken in air, the air convection effect was not avoided. For the bolometric sensing, the sensor was equipped in a vacuum, which further reduced the heat loss through air convection and led to much higher temperature increases.

### 3.4. Bolometer Performance

**Figure 5:**
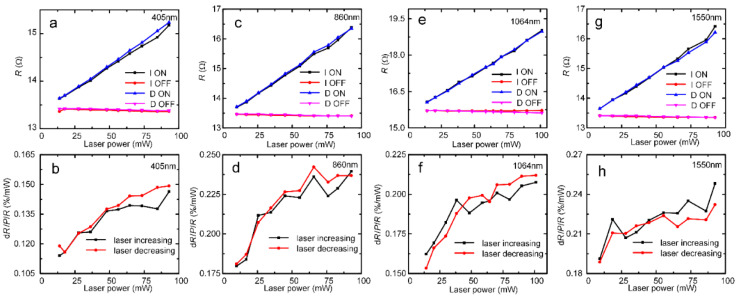
The *R*-*P* curves of the CNT–MoS_2_ composite network under laser irradiation at different wavelengths: (**a**) 405 nm; (**c**) 860 nm; (**e**) 1064 nm; (**g**) 1550 nm. The relative resistive responsivity per mW of power d*R*/(*P*·*R*) curves under laser irradiation at different wavelength: (**b**) 405 nm; (**d**) 860 nm; (**f**) 1064 nm; (**h**) 1550 nm.

The resistive bolometric response to the laser illumination in ultraviolet to near-infrared wavelength ranges was measured. Figure 4a shows the experimental setup. The details of the experiment can be found in the experimental section. We chose 405 nm, 860 nm, 1064 nm, and 1550 nm lasers, which can represent the UV–Vis–NIR range. The resistances when the laser is turned on and off is denoted as *R*_on_ and *R*_off_, respectively. The *R*_on_ and *R*_off_ results are summarized in Figure 5. Figure 5a shows the raw data for the resistance response of the CNT–MoS_2_ composite network to the 405 nm laser. In the first round of testing, the laser power increased from 14 mW to 93 mW. The corresponding *R*_on_ and *R*_off_ are denoted as I-ON and I-OFF, respectively, in the figure. When the laser was turned on, R showed a liner increasing trend as the laser power increased (I-ON). To study the repeatability of the response, the resistance response was measured again as the laser power was reduced from 93 mW to 14 mW. The corresponding *R*_on_ and *R*_off_ are denoted as D-ON and D-OFF, respectively, in the figure. As is shown in Figure 5a, the data of the decreasing round shows good consistency with that of the increasing round. This proves that the bolometric response of the composite network has good repeatability. The relative resistive responsivity per mW of power d*R*/(*P*·*R*) is calculated using (*R*_on_ − *R*_off_)*/*(*R*_off_**·***P*), where P is the incident laser power [1]. As shown in Figure 5b, as the incident laser power increases from 14 mW to 93 mW, the corresponding responsivity changes from 0.114%*/*mW to 0.149%*/*mW.

In addition, Figure 5c,e,g show the original data for the resistance responses to the 860 nm, 1064 nm, and 1550 nm lasers, respectively. All of the resistance responses show a similar trend, whereby the resistance increases linearly with the increasing laser power. The two rounds of data show good repeatability. Figure 5d,f,h show the relative resistive responses to the 860 nm, 1064 nm, and 1550 nm lasers, respectively. To check the bolometric response to lower laser power densities, the laser power was further reduced to 3 mW. Figure S5 shows that the CNT–MoS_2_ composite network also exhibits good repeatability and high responsivity to low laser powers of 3–20 mW. It should be noted that the *R*-*P* curve appears to be linear in Figure 5. However, the *R*-*P* curves were measured under lower laser power. As can be seen in Figure S5, the d*R*/d*P* value under the lower laser power is lower than that under higher laser power. Under higher laser power, the d*R*/d*P* values of the 405 nm, 860 nm, 1064 nm, and 1550 nm lasers are 0.0192 Ω/mW, 0.0319 Ω/mW, 0.0335 Ω/mW, and 0.0321 Ω/mW, respectively. However, under the lower laser power, the dR/dP values of the 405 nm, 860 nm, 1064 nm, and 1550 nm lasers are 0.0150 Ω/mW, 0.0156 Ω/mW, 0.0208 Ω/mW, and 0.0178 Ω/mW, respectively. This indicates that the dependence of the network resistance on the laser power is nonlinear.

**Figure 6:**
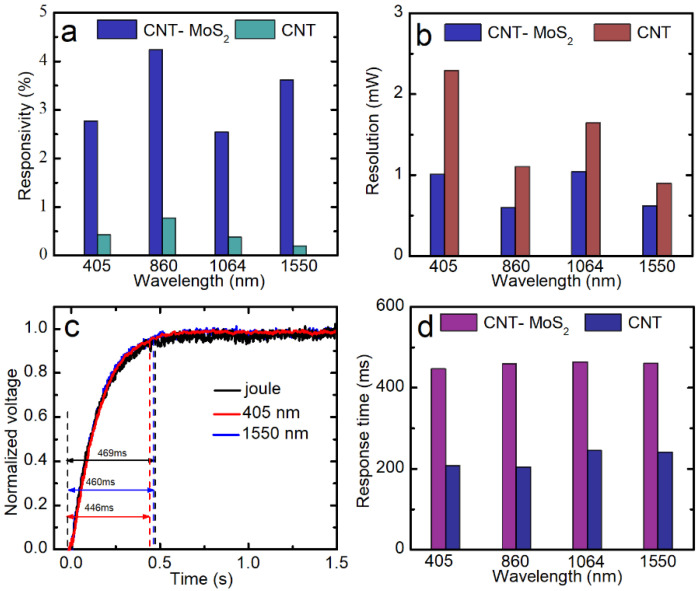
(**a**) A comparison of the reponsivity (d*R*/*R*) values of the unannealed CNT network and the CNT–MoS_2_ composite network under the same 2 mW/mm^2^ laser power density at different wavelengths. (**b**) A comparison of the sensitivity (the minimum detectable laser power) levels of the unannealed CNT network and the CNT–MoS_2_ composite network to different laser wavelengths. (**c**) Comparison of normalized voltage–time profiles between the modulated laser heating and joule heating, showing the same response times and confirming that the photoresponse to the laser is a bolometric effect. (**d**) A comparison of the response times of the unannealed CNT network and the CNT–MoS_2_ composite network to different laser wavelengths.

For better comparison of the CNT–MoS_2_ composite network and the CNT network, the responsivity is calculated by d*R*/*R* = (*R*_on_ − *R*_off_)/*R*_off_. For comparison, Figure S6 shows the *R*-*P* curves of the unannealed CNT network at different wavelengths. The annealed CNT network was also tested for comparison. However, the annealed CNT network showed very poor repeatability and responsivity, probably due to the unstable intertube connection. Thus, the unannealed CNT network was chosen for the bolometric performance study. Their responses for a laser power density of 2 mW/mm^2^ are compared and summarized in Figure 6a (raw data presented in Figures S7 and S8). It can be seen that the responsivity of the CNT–MoS_2_ composite network is much higher than that of the unannealed CNT network, which improves by 6.5-, 5.5-, 6.5-, and 18.5-fold under the 405 nm, 860 nm, 1064 nm, and 1550 nm laser irradiation, respectively.

In addition, it can be observed that when the laser is turned off, the variation of resistance (I-OFF and D-OFF) of the CNT–MoS_2_ composite network is small (less than 0.147%). The noise of the resistance readout is calculated to be as low as *R*_N_ = 0.02 Ω (the standard deviation of the I-OFF and D-OFF). By taking the resistance stability into consideration, the minimum detectable laser power can be calculated by *R*_N_/(dR/dP). Therefore, laser powers as low as 1.005 mW from the 405 nm laser, 0.592 mW from 860 nm, 1.040 mW from 1064 nm, and 0.616 mW from 1550 nm can be detected by the CNT–MoS_2_ composite network-based bolometer. For comparison, for the unannealed CNT network bolometer, the minimum detectable laser power is 2.287 mW from the 405 nm laser, 1.100 mW from 860 nm, 1.645 mW from 1064 nm, and 0.894 mW from 1550 nm. As summarized in Figure 6b, the minimum detectable laser power of the CNT–MoS_2_ composite network is lower than the unannealed CNT network, which indicates that the composite network-based bolometer shows higher detecting sensitivity.

Considering that the mechanism of the photoresponse could be photovoltaic or bolometric, to clarify the mechanism of the photoresponse, the resistance responses to the laser heating and the joule heating were compared. The experimental setup is shown in Figure 4a. To measure the transient resistive responses to the laser irradiation, the 405 nm, 860 nm, 1064 nm, and 1550 nm lasers were used as the optical sources. The laser output was modulated to a 0.2 Hz square wave using a function generator. In the comparative experiment, to measure the transient resistive response to the joule heating, a square-wave current of 16 mA in amplitude and 0.2 Hz in frequency was applied to the sample to check its response and to compare it with the response to the modulated laser. The transient voltage response (*V*-*t* profiles) was measured using the oscilloscope. The detailed raw data can be found in Figures S9 and S10 in the Supporting Information. All of the voltages of the CNT–MoS_2_ composite network increase and reach the steady state under the three scenarios (Figure S11).

Figure 6c shows the normalized voltage−time profiles (V*-*t*). Excellent agreement can be seen between the transient responses to the laser illumination and joule heating, where similar characteristic times of 469 ms for the joule heating, 446 ms for the 405 nm laser, and 460 ms for the 1550 nm laser heating can be seen. These results demonstrate that the photoresponse behavior of the CNT–MoS_2_ composite network is bolometric [13]. Furthermore, the response time of the CNT–MoS_2_ composite network and the CNT network under laser irradiation ay different wavelengths were compared. As shown in Figure 6d, the response time of the CNT–MoS_2_ composite network is about twice of that of the CNT network. As discussed above, the α value of the CNT–MoS_2_ composite network was measured to be about half of the α of the unannealed CNT network. Considering the similar suspended lengths of the samples, as demonstrated in Equation (1), the response time of the one-dimensional heat conduction under uniform heating was only determined by α. Thus, the response time results were consistent with the measured α results for the two samples discussed above.

Therefore, the mechanism of the photoresponse for the CNT–MoS_2_ composite network can be described as follows. Under laser illumination, the photons are absorbed by the sample, which produces photoexcited carriers (excitons) [1]. Subsequently, the electron–phonon interaction leads to a fast transfer of the energy into the CNT lattice. Then, the temperature increase provides more phonon scattering opportunities for electrons, thereby increasing the electrical resistance of the sample. Therefore, the resistance of the CNT–MoS_2_ composite network increases with the laser power. According to the one-dimensional thermal conduction model shown in Equation (1), the decay time (the corresponding time when the normalized voltage reaches 0.95) can be derived as Δt_c_ = 0.2026L^2^*/*α. Thus, the response time is proportional to *L*^2^ and inversely proportional to α [1]. In our work, the suspended length of the CNT–MoS_2_ composite network was 2.15 mm. If the suspended length of the sample is reduced to 350 µm (the typical size of a pixel element of bolometric detector arrays) in the future, the response time of the CNT–MoS_2_ composite network will be reduced to 1/36 of the original response time, corresponding to 11.76–12.25 ms and a frame rate of 4150–4000 Hz, meeting the requirements for real-time infrared imaging.

## 4. Conclusions

In summary, MoS_2_ nanoflowers were composited with the MWCNT network via a facile self-assembling strategy to boost the bolometric performance. The α-*T* curve demonstrated that the MoS_2_ nanoflowers provide significant phonon scattering and affect the intertube interfaces, decreasing α by 51%. As the temperature increased from 296 K to 320 K, the relative TCR increased from 0.04%*/*K to 0.25%*/*K. The detection experiment under low laser power proved that the CNT–MoS_2_ composite network had strong sensitivity. It showed 5–18-fold enhancements in resistive responsivity compared with the pure CNT network to the 405–1550 nm laser irradiation at room temperature (RT). Under 2 mW/mm^2^ power density for the 1550 nm laser, the responsivity reached 3.61%. The response time range of the 350-µm-long sample was about 11.76–12.25 ms, which was consistent with the joule heating result. This confirmed that the photoresponse of the CNT–MoS_2_ composite network was bolometric. The simple device structure and the removal of the requirement for high-quality CNTs represent steps forward towards the wide application of CNT-based IR detectors.

## Figures and Tables

**Table 1:** Different concentrations of precursors used for synthesizing the CNT–MoS_2_ composite network.

Solution	Na_2_MoO_4_ 2H_2_O (g)	CH_4_N_2_S (g)	DI Water (mL)
1	0.1210	0.1142	30
2	0.2420	0.2284	30

**Table 2:** Details of the samples measured in this study.

Sample	Length (mm)	Width (mm)	Thickness (μm)	Density (kg∙m^−3^)
S1 (Low MoS_2_ composite density)	5.74 ± 0.01	0.68 ± 0.01	33 ± 2	1470 ± 50
S2 (High MoS_2_ composite density)	6.90 ± 0.01	0.76 ± 0.01	36 ± 2	1889 ± 50
Unannealed CNT Network	6.49 ± 0.01	0.73 ± 0.01	21 ± 2	702 ± 50
Annealed CNT Network	6.49 ± 0.01	0.45 ± 0.01	21 ± 2	365 ± 50

## Data Availability

Not applicable.

## Supplementary Materials

The following supporting information can be downloaded at: https://www.mdpi.com/article/10.3390/nano12030495/s1 Figure S1. Measure the diameter of (a) 405 nm, (b) 860 nm, (c) 1064 nm, and (d) 1550 nm laser through knife edge technique [66]; Figure S2. Low- and High- magnification of SEM images of the sample 1 with low MoS_2_ decoration; Figure S3. Low resolution of SEM images of the CNT-MoS_2_ composite network; Figure S4. The measured *α* and resistivity of the composite network at different temperatures (296 K-320 K). The measurement uncertainty of *α* based on the TET technique is ±10%, which is omitted in the figure for better comparison; Figure S5. the *R*-*P* curve comparison of the CNT-MoS_2_ composite network under low laser power irradiation with different wavelength: (a) 405 nm, (b) 860 nm, (c) 1064 nm, (d) 1550 nm and (e) the comparison of the dR/R-PD (power density) curve; Figure S6. the *R*-*P* curve of the pure CNT network with different wavelength: (a) 405 nm, (b) 860 nm, (c) 1064 nm, and (d) 1550 nm; Figure S7. the dR/R-PD (power density) curve of the pure CNT network under laser irradiation with different wavelength: (a) 405 nm, (b) 860 nm, (c) 1064 nm, and (d) 1550 nm; Figure S8. the dR/R-PD curve of the CNT-MoS_2_ composite network under laser irradiation with different wavelength: (a) 405 nm, (b) 860 nm, (c) 1064 nm, and (d) 1550 nm; Figure S9. The normalized voltage-time profiles of the pure CNT network under the laser irradiation of different wavelength: (a) 405 nm, (b) 860 nm, (c) 1064 nm, and (d) 1550 nm; Figure S10. The normalized voltage-time profiles of the CNT-MoS_2_ composite network under the laser irradiation of different wavelength: (a) 405 nm, (b) 860 nm, (c) 1064 nm, and (d)1550 nm; Figure S11. the voltage-time profiles of the composite network under the modulated laser heating and the joule heating. (With offset for comparison)
