# Supplementary material for: Preparation and Bolometric Responses of MoS2 Nanoflowers and Multi-Walled Carbon Nanotube Composite Network
Source: Nanomaterials (Basel). 2022 Jan 31;12(3):495. doi: 10.3390/nano12030495 (PMC8839724; doi:10.3390/nano12030495)
Supplement: Supplementary file 1 [file nanomaterials-12-00495-s001.zip › nanomaterials-1553878-supplementary.pdf]

nanomaterials

Supporting Information

Preparation and Bolometric Responses of MoS_2 Nanoflowers and Multi-Walled Carbon Nanotube Composite Network

Qin Wang, Yu Wu, Xin Deng, Liping Xiang, Ke Xu, Yongliang Li and Yangsu Xie *

College of Chemistry and Environmental Engineering, Shenzhen University, Shenzhen 518055, China; WQ1910223067@163.com (Q.W.); 2017145023@email.szu.edu.cn (Y.W.); dengxin2017@email.szu.edu.cn (X.D.); xlp727104513@163.com (L.X.); xkenergy@hotmail.com (K.X.); liyli@szu.edu.cn (Y.L.)

* Correspondence: ysxie@szu.edu.cn

Measurement of Laser diameter

The laser power density is calculated by $P/(\pi d^2/4)$, where P is the laser power, d is the laser beam diameter. The P is measured by optical power meter from Thorlabs company. The laser beam diameter is measured through knife-edge technique.[1] During the measurement, the laser beam can be seen a radially symmetric Gaussian beam and the beam radius is measured at the position corresponding to the intensity drop to $1/e^2$. And the d is calculated by d= $2\sqrt{2}[0.552(x_{10}-x_{90})]$, where x_{10} and x_{90} are the beam intensity falls to 10% and 90% of the full value. The results are shown in Figure S1, the laser beam diameter is 3.74 mm, 3.67 mm, 3.20 mm, 3.28 mm for 405 nm, 860 nm, 1064 nm and 1550 nm, respectively.

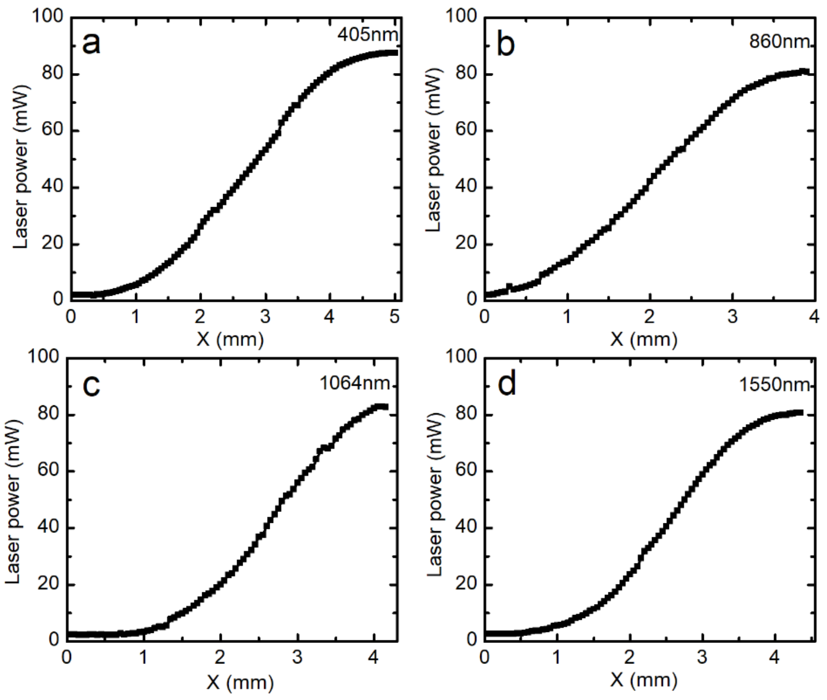

Figure S1. Measure the diameter of (**a**) 405 nm, (**b**) 860 nm, (**c**) 1064 nm, and (d) 1550 nm laser through knife edge technique.[1]

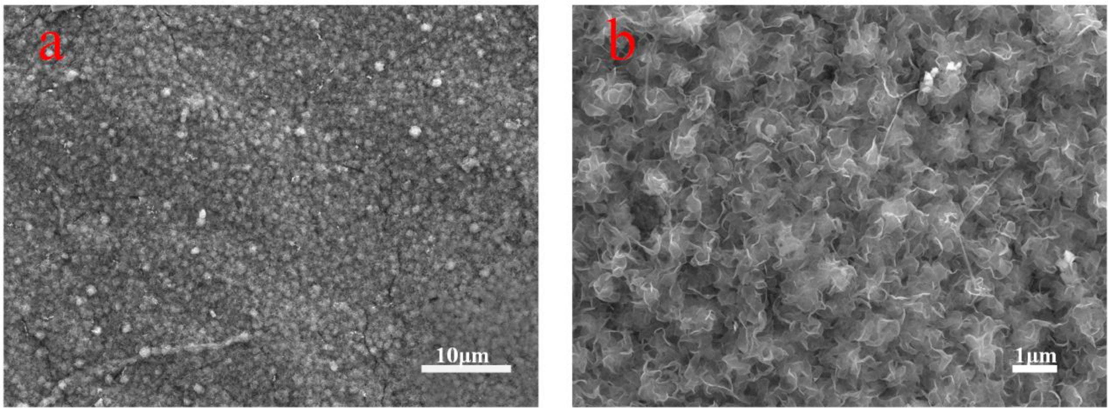

Figure S2. Low- and High- magnification of SEM images of the sample 1 with low MoS_2 decoration.

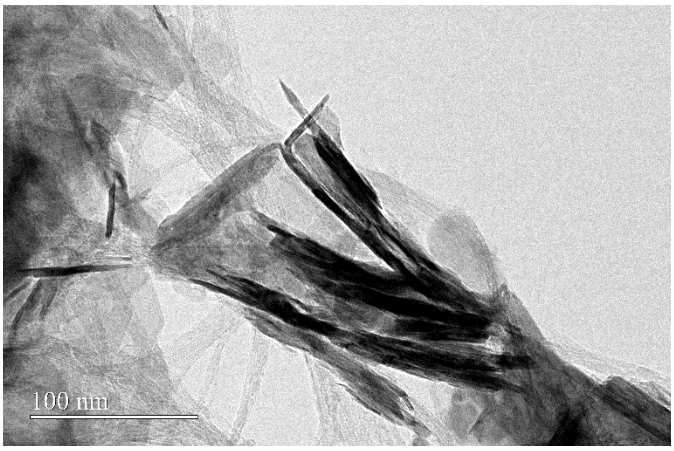

Figure S3. Low resolution of SEM images of the CNT-MoS_2 composite network.

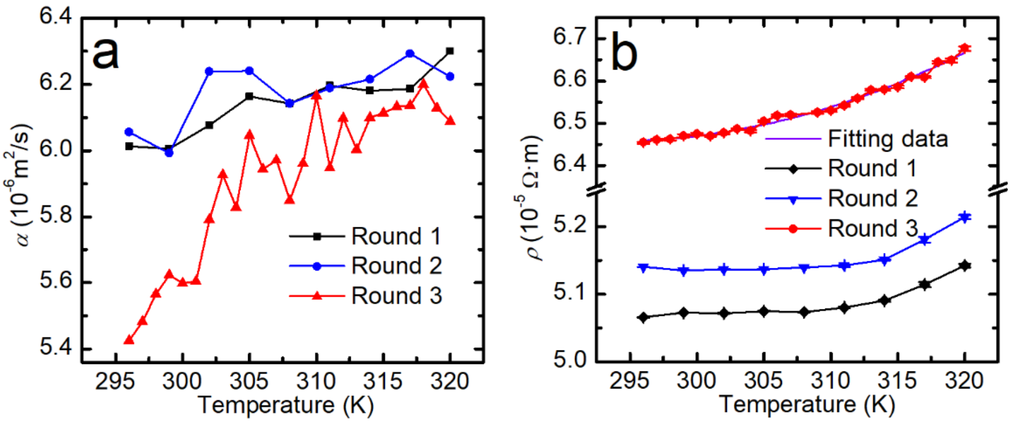

Figure S4. The measured α and resistivity of the CNT-MoS_2 composite network at different temperatures (296 K-320 K). The measurement uncertainty of α based on the TET technique is ±10%, which is omitted in the figure for better comparison.

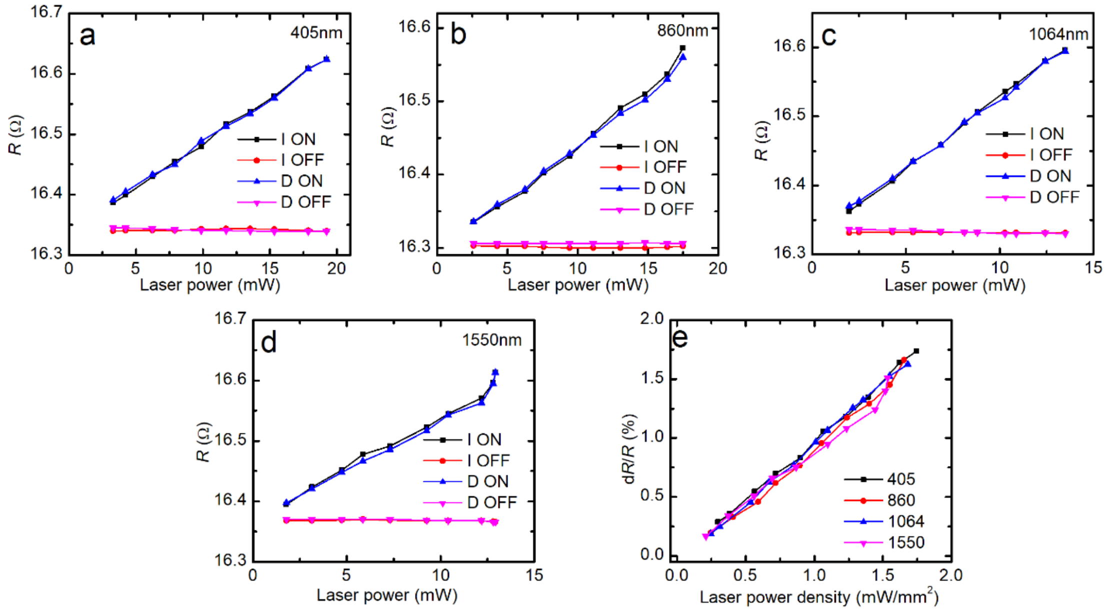

Figure S5. the *R*-*P* curve comparison of the CNT-MoS_2 composite network under the low laser power irradiation with different wavelength: (**a**) 405 nm, (**b**) 860 nm, (**c**) 1064 nm, (**d**) 1550 nm and (**e**) the comparison of the dR/R-PD (power density) curves under the laser irradiation of four different wavelengths.

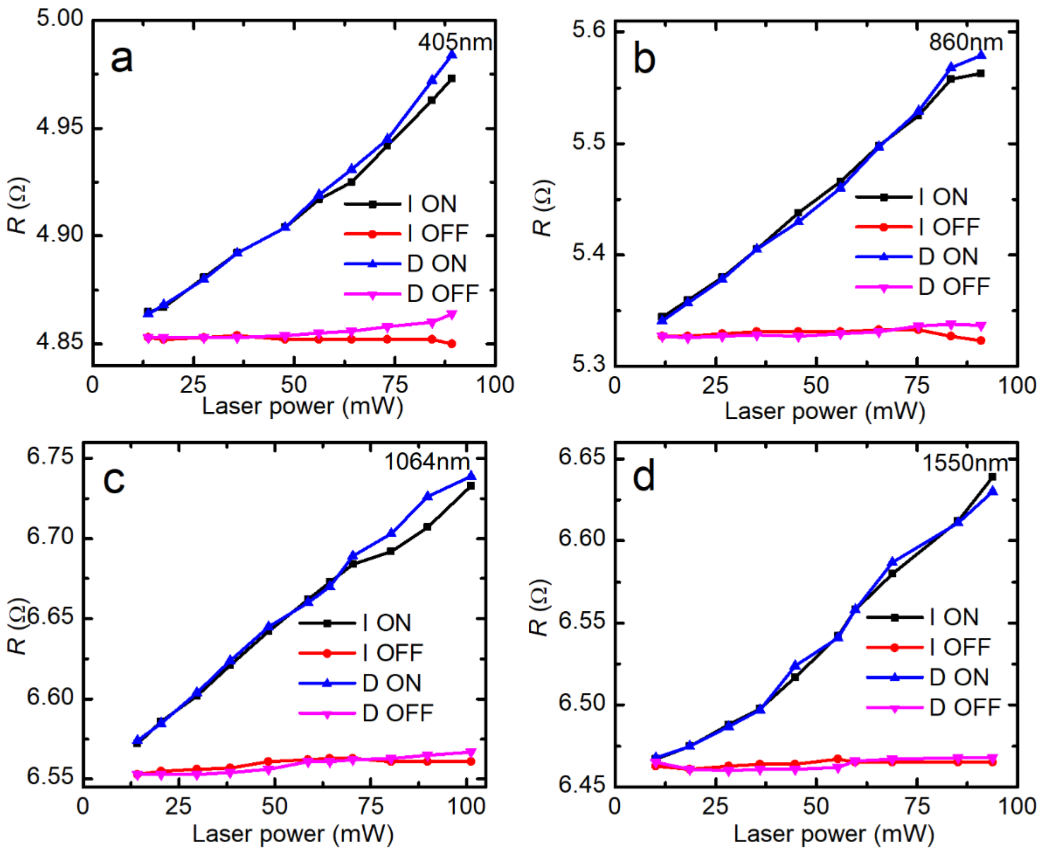

Figure S6. the *R-P* curve of the pure CNT network with different wavelength: (**a**) 405 nm, (**b**) 860 nm, (**c**) 1064 nm, and (**d**) 1550 nm.

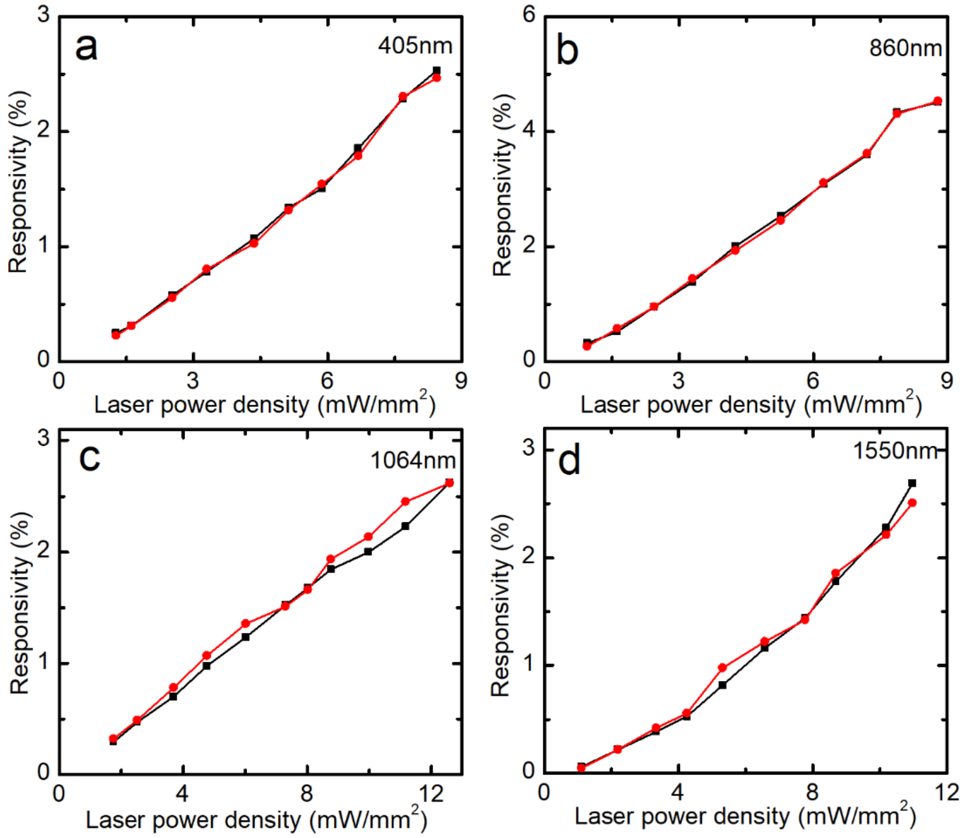

Figure S7. the dR/R-PD (power density) curve of the pure CNT network under laser irradiation with different wavelength: (**a**) 405 nm, (**b**) 860 nm, (**c**) 1064 nm, and (**d**) 1550 nm.

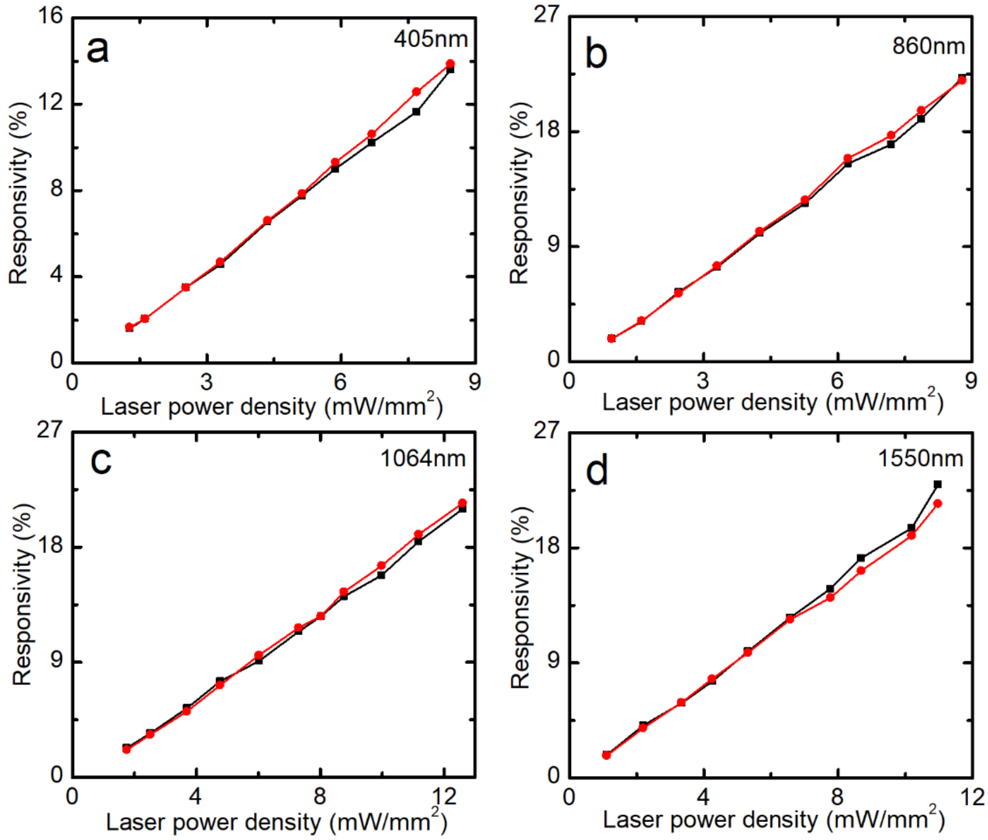

Figure S8. the dR/R-PD curve of the CNT-MoS_2 composite network under laser irradiation with different wavelength: (**a**) 405 nm, (**b**) 860 nm, (**c**) 1064 nm, and (**d**) 1550 nm.

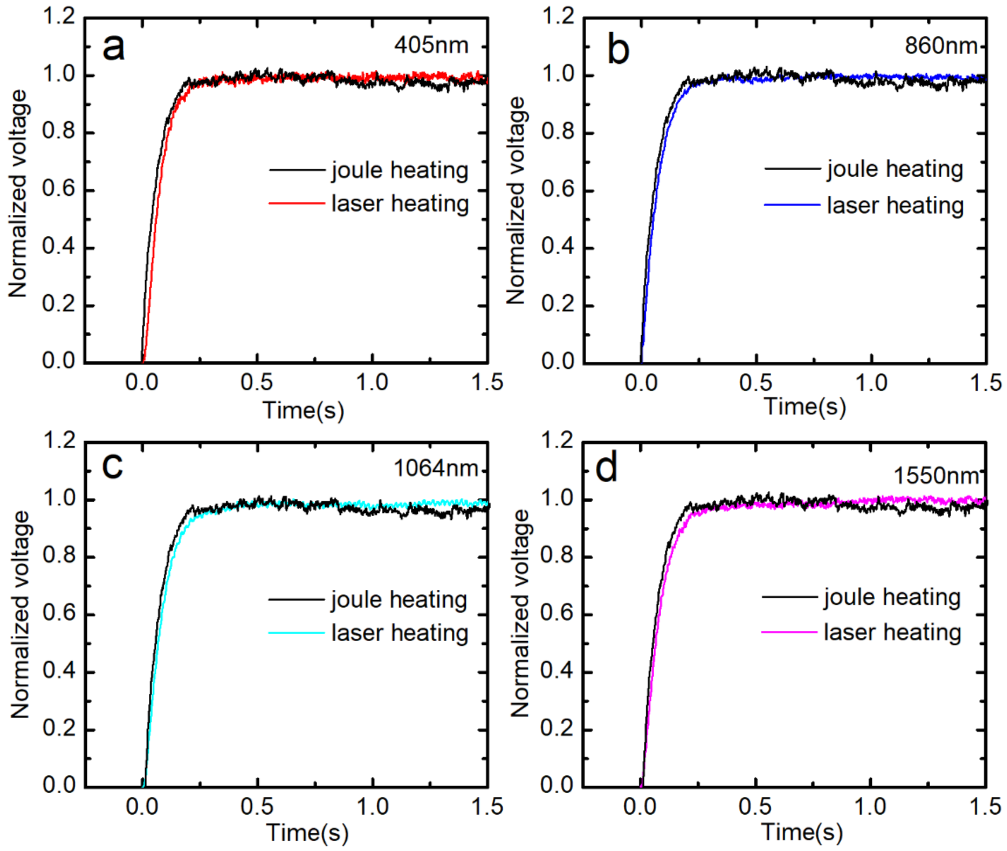

Figure S9. The normalized voltage-time profiles of the pure CNT network under the laser irradiation of different wavelength: (**a**) 405 nm, (**b**) 860 nm, (**c**) 1064 nm, and (**d**) 1550 nm.

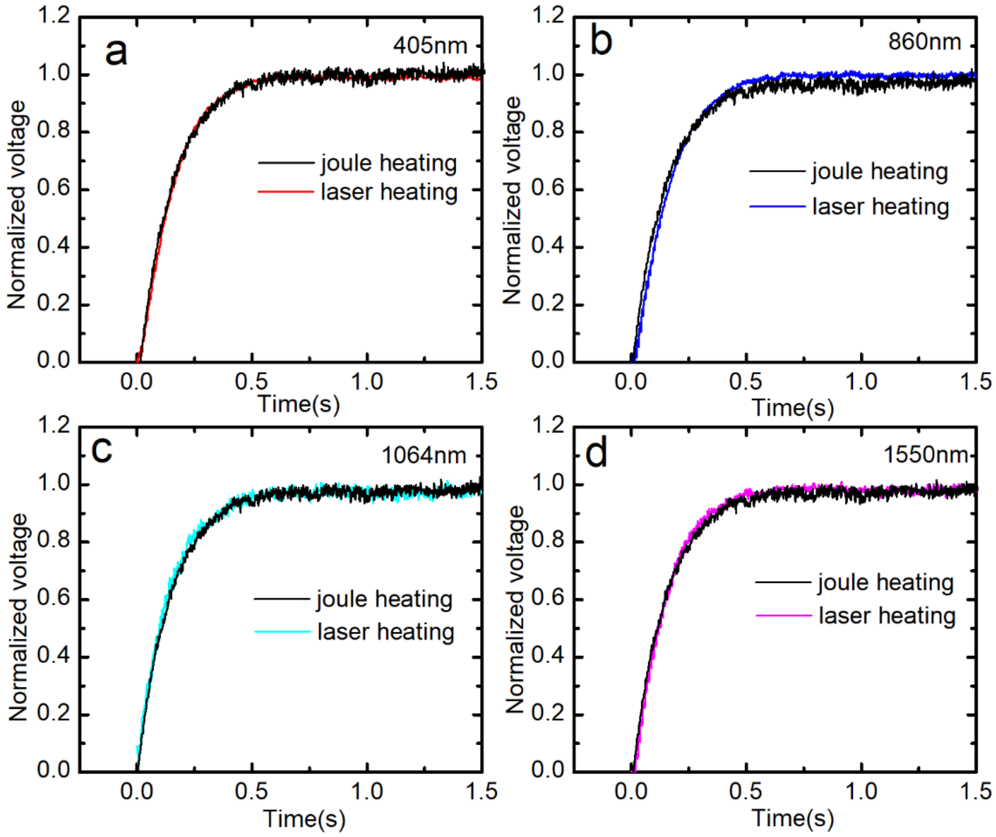

Figure S10. The normalized voltage-time profiles of the CNT-MoS_2 composite network under the laser irradiation of different wavelength: (**a**) 405 nm, (**b**) 860 nm, (**c**) 1064 nm, and (**d**)1550 nm.

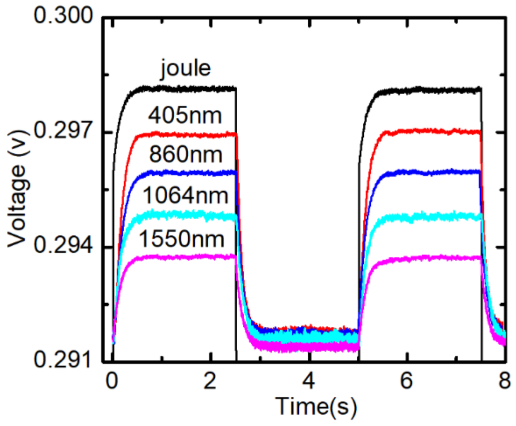

Figure S11. the voltage-time profiles of the composite network under the modulated laser heating and the joule heating. (With offset for comparison)

Reference

1. Khosrofian, J.M.; Garetz, B.A. Measurement of a Gaussian Laser-Beam Diameter through the Direct Inversion of Knife-Edge Data. *Applied Optics*, **1983**, *22*, 3406–3410.
